# Ongoing Pregnancies following Cosmetic Micromanipulation of Preimplantation Embryos in Patients with Implantation Failure

**DOI:** 10.1155/2015/734793

**Published:** 2015-10-13

**Authors:** Iman Halvaei, Mohammad Ali Khalili, Somayyeh Safari, Navid Esfandiari

**Affiliations:** ^1^Research and Clinical Center for Infertility, Shahid Sadoughi University of Medical Sciences, Bou-Ali Avenue, Safayeh, Yazd 89168-77391, Iran; ^2^Division of Reproductive Endocrinology and Infertility, Department of Ob-Gyn, Geisel School of Medicine at Dartmouth, Lebanon, NH, USA

## Abstract

Cosmetic micromanipulation is defined as fragment and coarse granulation removal from preimplantation embryos. We report two cases of pregnancies in patients with implantation failure following cosmetic micromanipulation.

## 1. Introduction

One of the most important factors involved in assisted reproduction technology (ART) success is the quality of embryo(s) generated in vitro [1, 2]. Embryos are routinely evaluated in an IVF lab and the best embryos are selected according to the morphology criteria on the day of embryo transfer (ET) [3, 4]. Although morphological evaluation has certain limitations, it has remained the most common method for embryo scoring [5, 6]. Presence of fragmentation in embryo has been considered an important parameter that affects morphology score and the extent of fragmentation is associated with the embryo viability. Fragments are referred to anuclear membrane-bound cytoplasmic structures seen between the blastomeres or between the blastomeres and zona pellucida (ZP) and resulted from cell divisions. It is estimated that about 40% of embryos generated in vitro show some extent of fragmentation during their first cleavage [7]. Fragmented embryos are associated with morphological hallmarks of apoptosis, and it is proposed that fragmentation may result from program cell death activation in some blastomeres [8]. The degree of fragmentation is generally categorized to mild (<10%), moderate (10–25%), and severe when more than 25% of the embryo is occupied by fragments. A great deal of efforts have been made to improve the quality of the embryos generated in vitro using advanced techniques such as embryo defragmentation [9]. Since the best embryos are selected based on their morphological features, a question remains as to whether ART outcomes improve, if a poor looking embryo is refurbished into a good looking one.

Beside cytoplasmic fragmentation which is cornerstone of each embryo grading system, another dysmorphism of embryo is the presence of coarse granulation around the blastomeres which mainly originated from perivitelline space (PVS) debris before embryo formation. It is suggested that the source of debris in PVS might be ooplasmic or remnants of coronal cells [10, 11]. In this case series, we sought to evaluate the effect of cytoplasmic fragment removal and coarse granulation removal from PVS (cosmetic micromanipulation) at the cleavage stage embryo before embryo transfer on pregnancy outcomes.

## 2. Controlled Ovarian Hyperstimulation

Controlled ovarian hyperstimulation was done by Karma/Gonal F 150 IU/day administration commencing on day two of menstrual cycle. When at least one follicle reached ≥14 mm in diameter, 0.25 mg GnRH antagonist (Cetrotide, Merck Serono, Germany) was started and continued until the hCG injection day. Ovarian response was checked by serial transvaginal ultrasound and serum estradiol levels. When the patient had at least two 17 mm follicles, 10000 IU hCG (Pregnyl, Organon, Netherlands) was administrated followed by an oocyte retrieval 34–36 hr later. The fertilization was checked after 16–18 hrs and embryo culture was done using G-1 v5 (Vitrolife, Sweden) until day three.

## 3. Cosmetic Micromanipulation

**Figure 1:**
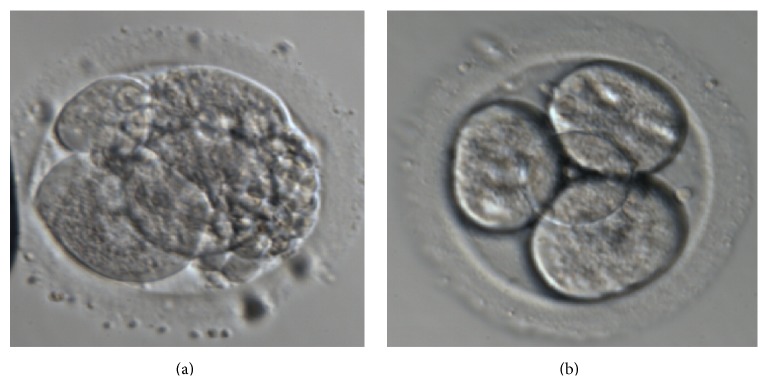
Cosmetic micromanipulation on cleavage stage embryo. (a) Embryo before cosmetic micromanipulation; (b) embryo after cosmetic micromanipulation.

The fragmented embryos that were selected for ET were incubated in Ca-Mg-free culture media for five min. Fragment removal was done using a micropipette (inner diameter: 10–12 *μ*m) and holding micropipette (inner diameter: 120–150 *μ*m). The fragment removal micropipette was filled with PVP 40% and then mineral oil. The tip of micropipette was then washed in Ca-Mg-free microdroplets. The embryo was rotated by micropipettes for better orientation of fragments. Then, the embryo was held by holding pipette and ZP was hatched at 3 o'clock position by 1480 nm wave length infrared diode laser for two msec duration to open a 10–12 *μ*m hole in ZP, and the operation was traced with a video monitor. The micromanipulation micropipette was entered from ZP hole and the fragments were gently removed from embryo (Figure 1). For coarse granulation removal, the micropipette was gently moved closed to debris and granules around the blastomeres were aspirated into the micropipette. The embryo was carefully washed in G-1 v5 culture media and was cultured in the 37°C and 6% CO_2_ incubator until ET.

The ethics committee approved this study and signed written consent was obtained from the patients. A positive beta hCG confirmed chemical pregnancy that was checked after fourteen days. Implantation was defined as presence of a gestational sac. Clinical pregnancy was defined to detection of fetal heartbeat seven weeks after ET. Demographic and clinical data of cases are shown in Table 1.

## 4. Case 1

Case 1 was a 28-year-old patient with primary male factor infertility. Her husband had a history of varicocelectomy which had not improved the sperm parameters. Two embryos were subjected to cosmetic micromanipulation and seven embryos were cryopreserved. The cryopreserved embryos also had the same fragmentation percent and pattern. The patient had the history of four implantation failures and one fresh ET and three frozen-thawed ET cycles. There were no multinucleated blastomeres in embryos. Each embryo had circular shape and moderately even blastomeres on day three. Efforts were made to defragment the embryos as timely as possible to avoid long stay of embryo out of the incubator. The percent of fragmentations in first embryo and second embryo subjected to cosmetic micromanipulation were 15% and 20% with pattern of localized and scattered fragmentation, respectively. One embryo was implanted and the clinical pregnancy outcome was positive.

## 5. Case 2

This was a 36-year-old patient with primary male factor infertility. Seven eggs were retrieved and five mature oocytes underwent ICSI. Two embryos developed from three normally fertilized eggs. Two embryos were subjected to micromanipulation with two and four cells on day two with no blastomere multinucleation. Both embryos had circular shape, while one embryo had uneven blastomeres. The embryos had 20% and 40% fragmentation with localized and distributed patterns, respectively. The patient had the history of three ICSI fertilization failures and two implantation failures. The quality of embryos developed in this cycle was the same as her previous cycles. Coarse granulation removal was performed for this case. One embryo was implanted and the clinical pregnancy outcome was positive.

## 6. Discussion

To the best of our knowledge, this is the first report of clinical pregnancies following preimplantation embryo cosmetic micromanipulation (fragments and coarse granules removal). It was previously shown that both degree and pattern of fragmentation can affect the implantation potential of human embryos [9]. Presence of fragments can be scattered and in contact with several blastomeres, occupying the whole embryo, or be localized which is in contact with one blastomere [12]. A previous report has indicated that embryos with scattered fragments have better prognosis in comparison to embryos with localized fragments [9]. It is already known that implantation and pregnancy rates following ET of high fragmented embryos (over 25%) are disappointing [2, 13, 14]. Also, the total cell number of blastocysts that are developed from fragmented embryos has been reported to be lower [15].

One probable cause would be interfering of fragments with further embryo cleavage by presence in cleavage axis or obstruction of cell-cell communication, which is necessary for subsequent compaction and blastulation. It is accepted that cell-cell communication is essential for embryo compaction and fragments can theoretically interfere the blastomeres junction and therefore impair compaction process. An ultrastructural study however has shown that, with presence of fragments, blastomeres can contact each other from opposite site [16]. Theoretically, fragments removal can improve the viability of poor quality embryos by increasing the number of cell-cell contacts and restoring blastomeres relationship. Second, degeneration of neighboring blastomeres due to possible toxic microenvironment produced by fragments may be overcome by defragmentation. Swelling and lysis of fragments might generate toxic microenvironment inside the ZP cavity, which would be detrimental for neighboring blastomeres [17, 18]. Our investigation is an ongoing case-control study that aims to assess whether refurbishing poor looking embryos results in better implantation outcomes.

Some believe that large fragments may contain essential organelles, such as mitochondria, and removing large fragments in early stages of development can deprive embryo from such essential organelles in next stages of development [9]. Size of fragments seems to be important as to whether they have important cell contents or they are organelle-free with no biological efficacy. On the other hand, fragments removal in embryos with 0–15% or >35% fragmentation has been associated with no improvement in clinical outcomes [17]. It seems in severe fragmentation that fragments removal can only improve the look of the embryo and has no other beneficial effects on embryo quality and implantation outcome, because these embryos are usually associated with other abnormalities with compromised embryo viability.

In a large prospective study, it was shown that blastulation rate increased following fragment removal compared to the control group, while apoptotic index decreased [19]. Debris in PVS is one of extracytoplasmic dysmorphisms of the mammalian oocytes [20]. Ultrastructural studies have shown that these structures are extracellular matrix that consisted of granules and filaments between oolemma and ZP, or remnants of coronal cell processes which pass through ZP and reach oolemma [10, 11]. One of the suggested causes for PVS granularity would be related to exocytosis of cortical granules. It was shown that 15% of meiotically mature human oocytes show signs of incomplete and premature exocytosis of cortical granules [21]. Also, Miao et al. (2009) showed that aging oocytes can result in premature exocytosis of cortical granules [22]. Animal studies have also shown that coarse granulation in PVS may be actually entrapped cumulus cells due to an abnormal ZP structure [23]. When the oocyte is fertilized and developed, these granularities can be found in subzona area next to the blastomeres. It was reported that presence of coarse granulation in PVS impairs the rates of implantation and pregnancy [24]. One specific goal in our study was to remove the subzona granularity as part of cosmetic micromanipulation per se. However, these coarse granulations may be attached to the ZP, requiring more manipulation to remove them from the embryos. In this study, we tried to remove the granulation which was loosely attached to ZP to avoid aggressive manipulation.

In conclusion, cosmetic micromanipulation on embryos can improve clinical pregnancy in patients with previous implantation failure. Well-designed randomized clinical trials are needed to answer the question of whether beautifying embryos after cosmetic micromanipulations is associated with better ART outcome. Further studies are also needed to elucidate the feasibility of this technique on frozen-thawed embryos in frozen-thawed ET cycles.

## Figures and Tables

**Table 1:** Laboratory and clinical data for cases with cosmetic micromanipulations.

Parameters	Case 1	Case 2
Male age	32	46
Duration of infertility	6	16
Sperm count (million/mL)	60 × 10^6^	5 × 10^6^
Sperm progressive motility (%)	33	10
Sperm normal morphology (%)	1	1
bFSH	4.5	8.6
LH	2.8	4.7
Retrieved oocytes	23	7
MII oocytes	22	5

Fertilized oocytes	9	3
Formed embryos	9	2
Transferred embryos	2	2

Implanted embryos	1	1
Clinical pregnancy	+	+
